# Accounting for measurement error in log regression models with applications to accelerated testing

**DOI:** 10.1371/journal.pone.0197222

**Published:** 2018-05-30

**Authors:** Robert Richardson, H. Dennis Tolley, William E. Evenson, Barry M. Lunt

**Affiliations:** 1 Department of Statistics, Brigham Young University, Provo, UT, United States of America; 2 Department of Physics, Brigham Young University, Provo, UT, United States of America; 3 Department of Information Technology, Brigham Young University, Provo, UT, United States of America; Politecnico di Milano, ITALY

## Abstract

In regression settings, parameter estimates will be biased when the explanatory variables are measured with error. This bias can significantly affect modeling goals. In particular, accelerated lifetime testing involves an extrapolation of the fitted model, and a small amount of bias in parameter estimates may result in a significant increase in the bias of the extrapolated predictions. Additionally, bias may arise when the stochastic component of a log regression model is assumed to be multiplicative when the actual underlying stochastic component is additive. To account for these possible sources of bias, a log regression model with measurement error and additive error is approximated by a weighted regression model which can be estimated using Iteratively Re-weighted Least Squares. Using the reduced Eyring equation in an accelerated testing setting, the model is compared to previously accepted approaches to modeling accelerated testing data with both simulations and real data.

## Introduction

Standard regression estimates of covariate parameters become biased when the explanatory variables are measured with error [1, 2]. A number of methods have been proposed to address the issue of measurement error in a variety of settings [3–6]. Among the most popular in laboratory settings is the York method [7, 8], which relies on the physics of the model and requires specification of the observational variance.

The log regression model defines a class of problems that occur frequently in the laboratory sciences. These models can be written in the form

$$\begin{array}{c} E\left(Y_{i}\right)=\exp\left(f\left(X_{i}|\theta \right)\right), \end{array}$$ (1)

where *Y*_1_, …, *Y*_*n*_ are observed responses, **X**_*i*_ = (*X*_1,*i*_, …, *X*_*p*,*i*_)′ for *i* = 1, …, *n* are explanatory variables and ***θ*** is a vector of parameters. One example of this is the log-linear model [9]. The stochastic component is often assumed to be multiplicative, resulting in log(*Y*_*i*_) = *f*(**X**_*i*_|***θ***) + *ε*_*i*_ where *ε*_*i*_ is random error and is typically modeled as a normal distribution with mean 0 and variance *σ*^2^. While making the assumption of multiplicative error is appropriate in many settings, this approach also provides bias on the original scale of the data as *E*(*Y*_*i*_) = exp(*f*(**X**_*i*_|***θ***) + .5*σ*^2^). One advantage of the multiplicative model is that it simplifies mathematically. An alternative assumption for the stochastic component is additive error, where *Y*_*i*_ = exp(*f*(**X**_*i*_|***θ***)) + *ε*_*i*_. These two approaches will yield differing parameter estimates.

The International Standard for the estimation of the archival lifetime of optical media, [10], outlines the procedure and assumptions for estimating lifetime of optical media by accelerated testing. This standard has been widely applied in deriving the lifetime estimates (LEs) for optical discs [11–13]. The procedure involves applying extreme values of Temperature (T) and Relative Humidity (RH) to optical media and fitting the data with the reduced Eyring equation, which fits into the log regression model framework. The estimated model is then extrapolated to determine the lifetime expectancy under normal conditions. Because this extrapolation extends significantly past the range where the treatments are applied, a small amount of bias will significantly alter the results, so making assumptions that may produce bias should be considered carefully. This bias may be present by failing to account for measurement error or by making poor assumptions about how the random error is included in the model, be it additive or multiplicative.

The purpose of this paper is to address the log regression model from Eq (1) in the case where the error is additive and the explanatory variables are observed with error and to specifcially show how it can be applied to reduce bias in parameter estimation in accelerating testing models. The proposed methodology will convert a log regression model with measurement error into a weighted regression model using a Taylor series expansion around the error terms, which can then be estimated using iterative re-weighting methods. The advantage of this approach is that it reduces bias in parameter estimates by accounting for correct sources of error while remaining relatively simple compared to other techniques of fitting non-linear models in the presence of measurement error.

Section 2 describes the proposed approach to fitting the log regression model in the presence of measurement error. Section 3 describes accelerated lifetime testing and the reduced Eyring equation and compares the proposed methodology with generally accepted methods of fitting the reduced Eyring equation in a simulation. Section 4 applies the methodology to both the example data set provided with the International Standard on fitting accelerated lifetime testing, the ISO/IEC 10995, as well as a Millenniata data set of accelerated lifetime testing of over 100 M-Disc DVDs.

## Methods

The log regression model with additive observational error and measurement error can be written as

$$\begin{array}{c} Y_{i}=\exp\left(f\left(X_{i}+\delta_{i}|\theta \right)\right)+\varepsilon_{i} \end{array}$$ (2)

where ***δ***_*i*_ = (*δ*_1,*i*_, …, *δ*_*p*,*i*_)′ is introduced as a normal measurement error vector with mean 0 and variance **V**. For this work it is assumed that **V** is a diagonal matrix with diagonal $\nu^{2}={\left(\nu_{1}^{2},\ldots ,\nu_{p}^{2}\right)}^{\prime }$.

**Theorem 1**. *The model in* Eq (2) *can be approximated as a weighted regression model of the form*

$$\begin{array}{c} \log\left(Y_{i}\right)\approx f\left(X_{i}|\theta \right)+{\left(\frac{d}{d\delta_{i}}f\left(X_{i}+\delta_{i}|\theta \right){|}_{\delta_{i}=0}\right)}^{\prime }\delta_{i}+\exp\left(-f\left(X_{i}|\theta \right)\right)\varepsilon . \end{array}$$

The proof of Theorem 1 is included in Appendix A.

This result can be used to convert a measurement error log regression model into a weighted regression model. The expected value of log(*Y*_*i*_) is *f*(**X**_*i*_|***θ***) and the variance is

$$\begin{array}{c} \text{Var}\left(\log\left(Y_{i}\right)\right)=\sum_{j=1}^{p}\left(\frac{d}{d\delta_{j,i}}f\left(X_{i}+\delta_{i}|\theta \right){|}_{\delta_{i}=0}\right)\nu_{j}^{2}+\exp{\left(-f\left(X_{i}|\theta \right)\right)}^{2}\sigma^{2}. \end{array}$$ (3)

Define *w*_1_, …, *w*_*n*_ be the inverse of these variance terms,

$$\begin{array}{c} w_{i}=\frac{1}{\text{Var}(\log\left(Y_{i}\right))}. \end{array}$$ (4)

The parameter set ***θ*** can be estimated as the values that minimize

$$\begin{array}{c} \sum_{i=1}^{n}w_{i}{\left(\log\left(Y_{i}\right)-f\left(X_{i}|\theta \right)\right)}^{2}. \end{array}$$ (5)

Details of building weighted regression models and using least squares to estimate the parameters can be found for linear models in [14] and for general non-linear problems in [15].

Iteratively Re-weighted Least Squares (IRLS) will be used to estimate the parameters [16]. This approach accounts for the fact that the parameters are used to calculate the weights, but the weights are needed to estimate the parameters. The general approach is described as follows:

1. Choose starting parameter estimate ***θ***^(0)^ and calculate the weights $w_{1}^{\left(0\right)},\ldots ,w_{n}^{\left(0\right)}$ using Eqs (3) and (4) and set a counter, *b* = 1.
2. Set $\sigma^{2}=\frac{1}{n-1}\sum_{i=1}^{n}{\left(Y_{i}-\exp\left(f\left(X_{i}|\theta^{\left(b-1\right)}\right)\right)\right)}^{2}$
3. Set ***θ***^(*b*)^ equal to the minimizer of Eq (5) using weights $w_{1}^{\left(b-1\right)},\ldots ,w_{n}^{\left(b-1\right)}$.
4. Calculate updated weights $w_{1}^{\left(b\right)},\ldots ,w_{n}^{\left(b\right)}$ using the updated parameter set ***θ***^(*b*)^.
5. Iterate steps 2 through 4 by incrementing *b* until some convergence criteria is met. A simple convergence criteria that could be used is ‖***θ***^(*b*)^ − ***θ***^(*b*−1)^‖ < .001.

## Accelerated testing

The ISO/IEC 10995 data analysis deals with the time to failure, measured in hours, for 20 samples of media at each of three stress treatments: (85°C, 85%RH), (85°C, 70%RH), (65°C, 85%RH), and the time to failure for 30 samples of media at the stress treatment (70°C, 75%RH). Ambient operating conditions are assumed to be (25°C, 50%RH), and interpolation procedures are outlined to adjust to other ambient conditions. The ISO/IEC 10995 procedure takes these 90 time to failure data points and reduces each of the four treatments to only the median time to failure values for analysis. Since the extrapolation is so far beyond the test conditions, we have studied how using just the treatment medians relates to results from analyzing the full data sets. We have also considered the fitting procedures from Section 2 to take into account measurement errors as well as correct specification of the nature of the observational errors in the time to failure.

The reduced Eyring equation used in modeling accelerating testing data is

$$\begin{array}{c} Y_{i}=A\exp\left(C/T_{i}+BH_{i})\right), \end{array}$$ (6)

where *Y*_*i*_ are the observed data i.e. time to failure measured in hours, *T*_*i*_ and *H*_*i*_ are temperature in Kelvins and relative humidity measurements, and *A*, *B*, and *C* are unknown parameters. Proper estimation of these unknown parameters is crucial to appropriately extrapolate the reduced Eyring equation from the conditions in the accelerated lifetime testing to standard conditions. Eq (6) is used to model time to failure as a function of temperature and relative humidity using laboratory data.

### Modeling approaches

Four different approaches will be used to compare analysis of accelerated testing data. This includes the ISO standard, a log-linear model, an additive error model, and finally an additive error model with measurement error in the predictors.

#### ISO standard

The standard method of modeling accelerated testing is according to ISO/IEC 10995. This method tests the product at 4 specific combinations of temperature and relative humidity, then fits a model to the logarithm of the medians of each of these 4 treatments. Let *Y*_*i*,*j*_ represent the *j*-th data point from the *i*-th temperature and relative humidity combination. Set *Z*_*i*_ to be equal to the median of *Y*_*i*,1_, *Y*_*i*,2_, …, *Y*_*i*,*n*_*i*__ where *n*_*i*_ is the number of observations in treatment *i*. The model fitted for the ISO/IEC 10995 is

$$\begin{array}{c} \log\left(Z_{i}\right)=\log\left(A\right)+C/T_{i}+BH_{i}+\varepsilon_{i}. \end{array}$$

The parameters are then estimated using regression of 1/*T*_*i*_ and *H*_*i*_ on log(*Z*_*i*_). This model assumes that the medians of the data satisfy all the assumptions for standard regression: the relationship between log(*Z*) and the predictors is linear, and that the errors are independent, normally distributed, with constant variance. This approach assumes that the stochastic error term for the observed median is multiplicative.

#### Log-linear model

A more standard statistical approach to estimating data using the reduced Eyring equation is to perform a regression directly on the logarithm of the individual measurements [9]. In this case, *Y*_*i*,*j*_ will be the *j*-th observation from treatment *i* and the model to fit is

$$\begin{array}{c} \log\left(Y_{i,j}\right)=\log\left(A\right)+C/T_{i}+BH_{i}+\varepsilon_{i,j}. \end{array}$$ (7)

This model now assumes that the data satisfy all the requirements for linear regression, which are again that the log(*Y*_*i*_) has a linear relationship with the predictors, and the errors in the model are independent, normally distributed, and have constant variance. Like the medians model, this log-linear model assumes that the error is multiplicative.

One feature of this model and the ISO standard is that the distribution of the time to failure data conditional on temperature and relative humidity is log-normal. A result of this is that the expected of value of the data is *E*(*Y*_*i*_) = *A* exp(*C*/*T*_*i*_ + *BH*_*i*_ + .5*σ*^2^). In other words the expected value of the data is the reduced Eyring equation from Eq (6) scaled by exp(.5*σ*^2^). For large variances of the errors, this may introduce a significant bias on the original scale of the data.

#### Additive error model

The ISO standard and log-linear model are the models most often used to fit the reduced Eyring equation. Based on the methodology from Section 2, we propose two additional approaches. The first allows the error term to be additive in the reduced Eyring equation, but still assumes the variables are measured without error. Theorem 1 can be applied with ***δ***_*i*_ = **0** for all *i*. IRLS will be used to estimate the parameters with weights according to Eqs (3) and (4) with *ν*_*j*_ = 0 for all *j*.

The approximate weighted regression model is

$$\begin{array}{c} \log\left(Y_{i,j}\right)=\log\left(A\right)+C/T_{i}+BH_{i}+\frac{\varepsilon_{i,j}}{A\exp(C/T_{i}+BH_{i})}. \end{array}$$

The weight for every observation in the *i*-th treatment group is then proportional to [*A* exp(*C*/*T*_*i*_ + *BH*_*i*_)]^2^. The previous two methods discussed assume that the variances of the log of the data are equal for all observations. This method assumes the variances are equal at the original scale of the data but different at the log scale. It also provides an unbiased estimate of the data, as *E*(*Y*_*i*_) = *A* exp(*C*/*T*_*i*_+*BH*_*i*_), which may be an improvement over the log-linear model in some cases.

#### Additive error model with measurement error

Another assumption made by all three of the models so far is that the values for temperature and humidity are measured with no experimental error. In reality, the best that researchers can hope for in measuring these values is small enough error to be considered “negligible”. In fact, if measurement errors are merely moderate or even large, the variation we see in the observations may be due as much to measurement error in the predictor variables as to random error. This is particularly problematic for accelerated testing since, by design, the failure model is to be extrapolated beyond the data. Failure to account for this measurement error can also affect the parameter estimates and can therefore affect the prediction of new observations.

Assume that *δ*_*T*,*i*,*j*_ and *δ*_*H*,*i*,*j*_ are the measurement errors in temperature and relative humidity respectively with means of zero and variances $\nu_{T}^{2}$ and $\nu_{H}^{2}$. Using Theorem 1, the approximate weighted linear model would be

$$\begin{array}{c} \log\left(Y_{i,j}\right)=\log\left(A\right)+C/T_{i}+BH_{i}+\delta_{T,i,j}C/T_{i}^{2}+\delta_{H,i,j}B+\frac{\varepsilon_{i,j}}{A\exp(C/T_{i}+BH_{i})}. \end{array}$$

Using this model we see that the expected value of the log of the data is *E*(log(*Y*_*i*,*j*_)) = log(*A*) + *C*/*T*_*i*_+*BH*_*i*_, and using Eq (3), the variance is

$$\begin{array}{c} \text{Var}\left(\log\left(Y_{i,j}\right)\right)=C^{2}\nu_{T}^{2}/T_{i}^{4}+B^{2}\nu_{H}^{2}+\frac{\sigma^{2}}{{\left(A\exp\left(C/T_{i}+BH_{i}\right)\right)}^{2}}. \end{array}$$ (8)

Weights can then be assigned according to Eq (4) and the IRLS procedure outlined in Section 2 would be used to estimate the parameters.

### Simulations

We simulate data using the reduced Eyring equation. When the error is additive, the form is

$$\begin{array}{c} Y_{i,j}=A\exp\left(C/\left(T_{i}+\delta_{T,i,j}\right)+B\left(H_{i}+\delta_{H,i,j}\right)\right)+\varepsilon_{i,j}, \end{array}$$ (9)

and when the error is multiplicative, the model form is

$$\begin{array}{c} \log\left(Y_{i,j}\right)=\log\left(A\right)+C/\left(T_{i}+\delta_{T,i,j}\right)+B\left(H_{i}+\delta_{T,i,j}\right)+\varepsilon_{i,j}, \end{array}$$ (10)

where, for both models, *ε*_*i*,*j*_ ∼ *N*(0, *σ*^2^), $\delta_{T,i,j}∼N\left(0,\nu_{T}^{2}\right)$, and $\delta_{H,i,j}∼N\left(0,\nu_{H}^{2}\right)$

Whether using Eqs (9) or (10), the simulated data is generated using the values log(*A*) = exp(−13.68), *B* = −.0422, and *C* = 8,485. These values have been chosen to reflect a possible model that could drive real-world results. Four data treatment groups were simulated with temperature values in °C of 65, 70, 85, and 85 respectively for each treatment group and relative humidity values in % RH of 85, 75, 85, and 70 respectively per treatment group with 20 observations in each group. 5 different settings are determined for simulation. These settings vary by whether the data are simulated using Eqs (9) or (10), whether or not there is measurement error, and by the ratio between observational error and measurement error. The simulation settings are summarized in the list below.

1. Simulated from Eq (9) with additive error, with *σ*^2^ = 10,000, $\nu_{T}^{2}=0$, and $\nu_{H}^{2}=0$.
2. Simulated from Eq (10) with multiplicative error, with *σ*^2^ = .0225, $\nu_{T}^{2}=0$, and $\nu_{H}^{2}=0$.
3. Simulated from Eq (9) with additive error, with *σ*^2^ = 10,000, $\nu_{T}^{2}=.5$, and $\nu_{H}^{2}=.1$.
4. Simulated from Eq (9) with additive error, with *σ*^2^ = 2,500, $\nu_{T}^{2}=.1$, and $\nu_{H}^{2}=.1$.
5. Simulated from Eq (9) with additive error, with *σ*^2^ = 40,000, $\nu_{T}^{2}=.1$, and $\nu_{H}^{2}=.1$.

We fit the resulting simulated data using the 4 methods described in Section 3.1: the ISO/IEC 10995 procedure of calculating the median for each treatment and fitting a linear model to the log medians (MED), taking the log of the data and fitting a linear model (LL), using Theorem 1 to weight the treatment groups and account for additive error *ε*_*i*,*j*_ (IRLS), and using the IRLS method while accounting for measurement error (IRLS-ME). Each of the different models makes different assumptions about the data and measurement variances. These assumptions are given in Table 1 where the untransformed observed data are denoted as *Y*_*i*,*j*_.

**Table 1:** Model assumptions. The assumptions implicit for each model.

Model	Assumption
MED	log(*Y*_*i*,*j*_) has equal variance for all *i*
LL	log(*Y*_*i*,*j*_) has equal variance for all *i*
IRLS	*Y*_*i*,*j*_ has equal variance for all *i*
IRLS-ME	No assumptions of equal variance

The goal of learning the parameters of the model is to extrapolate from the experimental conditions to ambient operating conditions, which are assumed to be a temperate 25°C and relative humidity of 50%. For each of the settings, 10,000 data sets are simulated and the prediction bias is calculated as the absolute difference between the prediction made from the model’s estimated values of *A*, *B*, and *C* and the prediction found when using the values of *A*, *B*, and *C* that were used to generate the data. Table 2 reports the average absolute prediction bias, measured in hours, of the 10,000 simulated data sets for all 4 models. Lower values mean less bias and a more accurate prediction.

**Table 2:** Simulation study prediction bias comparison. Average absolute prediction bias for the 10,000 data sets from each simulation setting and for each model.

Simulation Setting	MED	LL	IRLS	IRLS-ME
1	52,700	50,000	47,900	49,000
2	63,800	52,000	52,500	52,300
3	54,300	51,300	49,200	48,900
4	42,900	36,400	36,000	35,200
5	68,500	68,100	67,000	67,000

According to the results in Table 2, the ISO standard is more biased than any of the other models in all the settings. Also, note that accounting for measurement error in the model leads to a lower bias when the data are simulated using measurement error. In the multiplicative error data set (setting 2) the LL model matched the model that generated the data, but the IRLS-ME method was not much more biased. When measurement error was larger with respect to observational error, as in setting 4, the advantage of IRLS-ME over the IRLS model was greater. In contrast, when measurement error was reduced and observational error increased in setting 5, the advantage of IRLS-ME over IRLS was not as significant. This shows that accounting for measurement error may be more or less important depending on how much observational error there is.

The code used to create the simulated data sets is found in S1 File.

## Data

### ISO/IEC 10995 example data

**Fig 1:**
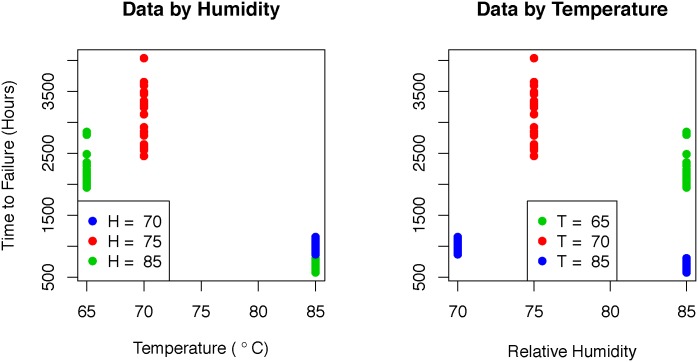
ISO example data. The data set shown is the ISO/IEC 10995 example data set. The left figure shows the data plotted by temperature and indexed by humidity and the right plot shows the data plotted by humidity and indexed by temperature. In both cases, the 4 treatments can be distinguished.

ISO/IEC 10995 includes an example data set simulated to show how to fit the median model, hereafter referred to as the example data set. These data are analyzed here using the four methods described in Section 3.1. The data are shown in Fig 1 and can be purchased with the international standard at http://www.iso.org/iso/catalogue_detail.htm?csnumber=56910. As with the simulated data above, there are 4 combinations of temperature and relative humidity, which we will refer to as treatments. The empirical variances for each treatment in the data set are shown in Table 3 with some rounding.

**Table 3:** Example data variances. The variance for each treatment at the original scale and the log scale for the example data set.

	Trtmt 1	Trtmt 2	Trtmt 3	Trtmt 4
Variance of *Y*_*i*,*j*_	188,400	60,300	7,100	4,600
Variance of log(*Y*_*i*,*j*_)	.0195	.0108	.0072	.0096

While neither of the rows are very uniform in variance, the change in variance in the log scale is much milder than the change in variance of the untransformed data. According to Table 1, this may suggest that the log-linear or median model fitting procedures may be appropriate. We also don’t know what measurement error may be present, because we did not simulate the data. For now, the IRLS-ME model assumes only a small amount of measurement error. Specifically, the IRLS-ME methods includes an error in temperature with a variance of.01, and an error in relative humidity with a variance of.25, which are reasonable values of measurement error variance on standard machines used for accelerated testing, perhaps even conservative in some cases. We cannot calculate the bias without knowledge of the parameters used to generate the data, but predictions of the lifetime of an object at 25° and 50% relative humidity are in Table 4 for each model, along with the 95% confidence interval width of this prediction. The 95% confidence intervals for the MED method are difficult to calculate using traditional theoretical variances of the estimates. Therefore, to be consistent, bootstrapping is used to calculate approximate confidence intervals for all 4 methods. Details on the bootstrapping procedure can be found in [16]. We also include a metric called Akaike’s information criterion (AIC). The metric is described in more detail in Appendix B, but it is a way of assessing how well the fitted model matches the data. A lower value of AIC implies that the model is better.

**Table 4:** Example data lifetime predictions. The prediction of lifetime in hours of an object in standard conditions, 95% confidence interval width, and AIC for the example data set.

Model	Prediction	CI Width	AIC
MED	331,700	190,300	−54.7
LL	340,800	132,000	−56.4
IRLS	322,600	159,200	−44.1
IRLS-ME	328,100	155,200	−56.4

While the IRLS-ME model has the lowest AIC, it is not different from the log-linear method and not very different from the median method. IRLS without measurement error is the worst of all the models. There is no way to tell which prediction is correct, and none seem to stand out as extreme or clearly wrong.

### Lunt data

**Fig 2:**
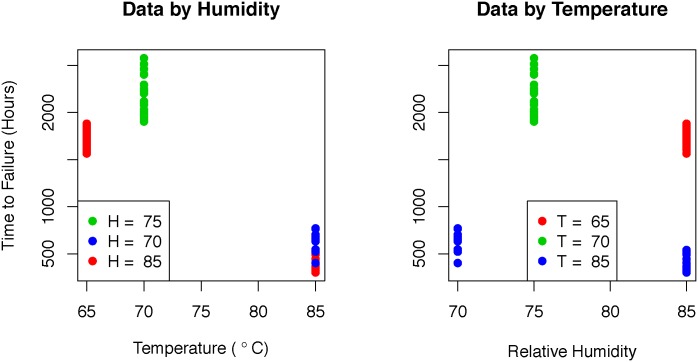
Lunt data set. Plotted is the Lunt data set. The left shows the data plotted by temperature and indexed by humidity and the right plot shows the data plotted by humidity and indexed by temperature.

In 2013, Millenniata tested over 100 M-Disc^®^ DVDs, following the ISO 10995 standard. The data gathered from this test were released to Barry Lunt, one of the authors. Herein this data set is referred to as the “Lunt” data. The Lunt data are shown in Fig 2 and is available in S2 File. Since this is not simulated data based on a fixed scenario, we do not know a priori what method to use. By looking at the variances, though, we can get an idea of which method may work better. The Lunt data variances for each treatment are in Table 5.

**Table 5:** Lunt data variances. The variance for each treatment at the original scale and the log scale for the Lunt data set.

	Trtmt 1	Trtmt 2	Trtmt 3	Trtmt 4
Variance of *Y*_*i*_	7,800	40,900	8,700	8,300
Variance of log(*Y*_*i*_)	.0026	.0083	.025	.049

Again, neither row is very uniform in variance, but as opposed to the example data set, the variances on the log scale are drastically different. This may suggest that the IRLS method may be the most appropriate model to fit. Again, we will assume that when the IRLS method is fitted with measurement error, the error in temperature has a variance of.01, and the error in humidity has a variance of.25. The predictions of the lifetime of an object at 25°C and 50% relative humidity are in Table 6 for each model, along with the confidence interval width and AIC.

**Table 6:** Lunt data lifetime predictions. The prediction of lifetime of an object in standard conditions, confidence interval width, and AIC for the Lunt data set.

Model	Prediction	CI Width	AIC
MED	363,200	610,200	−40.1
LL	367,900	266,800	−41.4
IRLS	421,500	306,400	−57.9
IRLS-ME	417,900	303,600	−48.6

Based on AIC, the best model for this scenario is the IRLS method without any measurement error. Also notable here is that the predicted values when using the IRLS or IRLS-ME methods for the extrapolation are far from the other two methods. While the median method and log-linear method had similar predictions and AICs, the confidence interval for the median method is much larger.

## Conclusion

Based on simulations, theory, and the analysis of real data, we have shown that there are some important matters to consider when fitting the reduced Eyring equation to data. When the variance is constant for the log data, then using a log-linear model may be appropriate. Even the median model, while it gave wider confidence intervals in general, seemed to give an unbiased estimate of the predictions when the log data had constant variance. However, when the log data do not have constant variance but the untransformed observations do, accounting for additive error using a Taylor series approximation and an IRLS fitting procedure may result in a better fit. When the data are somewhat in-between these two cases, where neither scale has variances that could be considered equal, the cause may be a small amount of measurement error, which may impact the model in a significant way.

When faced with a problem, a researcher should examine the variances of the two scales. Since none of these models are costly to fit, they all could be used and then the AIC’s could be compared to find a better model. A range of measurement error values could also be tried if the actual variances of the measurement error are not known, but often measurement error can be evaluated on the instruments prior to collecting the data. One of the most significant aspects of this study is that when real-world data are used, the variances were not equal in either the observation groupings or the log observation groupings, and the model fit using the IRLS model proved to be better. There may be a variety of other settings where this is the case.

The result in Theorem 1 may extend to other settings where the regression model variables are measured with error. For example, the Arrhenius equation is commonly used in chemistry and engineering to describe physical systems, or simple radioactive decay or growth models when extra variables affect the rate of decay or growth. The weighting method may also provide insight as to where observations may be placed to decrease the variance and increase the accuracy of prediction. Future work will explore these insights further.

## A Proof of Theorem 1

*Proof.* Let *g*(**δ**_*i*_, *ε*_*i*_) be equal to the log of Eq (2),

$$\begin{array}{c} g\left(\delta_{i},\varepsilon_{i}\right)=\log\left(Y_{i}\right)=\log\left(\exp\left(f\left(X_{i}+\delta_{i}|\theta \right)\right)+\varepsilon_{i}\right) \end{array}$$

The first order Taylor series approximation of *g*(**δ**_*i*_, *ε*_*i*_) about **δ**_*i*_ = **0** and *ε*_*i*_ = 0 is

$$\begin{array}{ccc} g(\delta_{i},\varepsilon_{i}) & \approx & g\left(\delta_{i},\varepsilon_{i}\right){|}_{\delta_{i}=0,\varepsilon_{i}=0}+{\left(\frac{d}{d\delta_{i}}g\left(\delta_{i},\varepsilon_{i}\right){|}_{\delta_{i}=0,\varepsilon_{i}=0}\right)}^{\prime }\delta_{i}+\left(\frac{d}{d\varepsilon_{i}}\left(\delta_{i},\varepsilon_{i}\right){|}_{\delta_{i}=0,\varepsilon_{i}=0}\right)\varepsilon_{i} \end{array}$$

The derivative of *g* with respect to **δ**_*i*_ is

$$\begin{array}{ccc} \frac{d}{d\delta_{i}}g\left(\delta_{i},\varepsilon_{i}\right) & = & \frac{1}{\exp\left(f\left(X_{i}+\delta_{i}|\theta \right)\right)+\varepsilon_{i}}\exp\left(f\left(X_{i}+\delta_{i}|\theta \right)\right)\left(\frac{d}{d\delta_{i}}f\left(X_{i}+\delta_{i}|\theta \right)\right) \end{array}$$

When **δ**_*i*_ and *ε*_*i*_ are set to 0, the derivative is

$$\begin{array}{ccc} \frac{d}{d\delta_{i}}g\left(\delta_{i},\varepsilon_{i}\right){|}_{\delta_{i}=0,\varepsilon_{i}=0} & = & \frac{d}{d\delta_{i}}f\left(X_{i}+\delta_{i}|\theta \right){|}_{\delta_{i}=0} \end{array}$$

The derivative of *g* with respect to *ε*_*i*_ is

$$\begin{array}{ccc} \frac{d}{d\varepsilon_{i}}g\left(\delta_{i},\varepsilon_{i}\right) & = & \frac{1}{\exp\left(f\left(X_{i}+\delta_{i}|\theta \right)\right)+\varepsilon_{i}} \end{array}$$

When **δ**_*i*_ and *ε*_*i*_ are set to 0, the derivative is

$$\begin{array}{ccc} \frac{d}{d\varepsilon_{i}}g\left(\delta_{i},\varepsilon_{i}\right){|}_{\delta_{i}=0,\varepsilon_{i}=0} & = & \exp(-f\left(X_{i}|\theta \right)) \end{array}$$

Combining these results leaves us with the desired approximation.

## B Assessing model fit

A metric for assessing quality of results from several different fitting models needs to respond to the accuracy of the estimated parameters, the accuracy of predictions, and the uncertainties. In addition, we expect the fitted model to have the ability to regenerate data with properties close to those of the original data set. Then if a particular model is fitted and the estimated parameters are used in a simulation to generate new data, we should see that the new simulated data and the original data are clearly consistent with one another. If the fitted model is unable to regenerate simulated data which is reasonably similar to the actual data, we can typically conclude that the model fit is poor or inadequate.

One metric used to assess a model fit’s quality of results is Akaike’s Information Criterion (AIC). The formula for the AIC is

$$\begin{array}{c} AIC=-2l(x;\hat{\theta })+2k\;, \end{array}$$

where $l(x;\hat{\theta })$ is the log likelihood of the data for a particular choice of likelihood function and estimated parameters $\hat{\theta }$, and *k* is the number of parameters. This value accounts both for the likelihood of the data for given fitted parameters and for the decrease in discrimination among models as the number of parameters increases. AIC as defined is generally negative, so the more negative AIC the greater expectation of quality of fit. Furthermore, a model with a smaller AIC (i.e. more negative) is more likely to be able to regenerate data similar to those used to fit the model. AIC is used as a quality metric in this paper, and in all cases when multiple procedures are used to fit the same data, the model with a smaller AIC will be considered to be more reliable than the others.

For this paper, the AIC was calculated using the maximum likelihood estimates of the parameters. For the MED and LL models, the likelihood function of the log data is a normal distribution with a mean of log(*A*) + *C*/*T*_*i*_+*BH*_*i*_ and a variance of *σ*^2^. For the IRLS method, the likelihood function is a normal with mean log(*A*) + *C*/*T*_*i*_+*BH*_*i*_ and a variance of ${\left(\frac{\sigma }{A\exp(C/T_{i}+BH_{i})}\right)}^{2}$. The mean for the IRLS-ME method is the same as the others, but the variance is $C^{2}\nu_{T}^{2}/T_{i}^{4}+B^{2}\nu_{H}^{2}+\frac{\sigma^{2}}{{\left(A\exp\left(C/T_{i}+BH_{i}\right)\right)}^{2}}$. Using the likelihood for the log data instead of the raw data will change AIC values, but it will not change the ordering, meaning that a lower AIC on the log data will still have a lower AIC for the raw data.

## Supporting information

S1 File Simulated data. R file containing information needed to reproduce simulated data sets from an Arrhenius equation, both with additive and multiplicative observation error and with or without measurement error.
(R) Click here for additional data file.

S2 File Lunt data. CSV file containing the actual data from an accelerated testing experiment performed by co-author Barry M. Lunt.
(CSV)
